# The genetic regulation of the terminating phase of liver regeneration

**DOI:** 10.1186/1476-5926-11-3

**Published:** 2012-11-20

**Authors:** Ingvild E Nygård, Kim E Mortensen, Jakob Hedegaard, Lene N Conley, Trine Kalstad, Christian Bendixen, Arthur Revhaug

**Affiliations:** 1 Department of Digestive Surgery, University Hospital of Northern-Norway, Tromsø 9038, Norway; 2 Department of Genetics and Biotechnology, Faculty of Agricultural Sciences, University of Aarhus, Tjele, DK-8830, Denmark; 3 Laboratory of Surgical Research, Institute of Clinical Medicine, University of Tromsø, Tromsø, 9037, Norway

**Keywords:** Angiogenesis, Apoptosis, Cell cycle, Microarray analysis, Partial hepatectomy, Porcine genes

## Abstract

**Background:**

After partial hepatectomy (PHx), the liver regeneration process terminates when the normal liver-mass/body-weight ratio of 2.5% has been re-established. To investigate the genetic regulation of the terminating phase of liver regeneration, we performed a 60% PHx in a porcine model. Liver biopsies were taken at the time of resection, after three weeks and upon termination the sixth week. Gene expression profiles were obtained using porcine oligonucleotide microarrays. Our study reveals the interactions between genes regulating the cell cycle, apoptosis and angiogenesis, and the role of Transforming Growth Factor-β (TGF-β) signalling towards the end of liver regeneration.

**Results:**

Microarray analysis revealed a dominance of genes regulating apoptosis towards the end of regeneration. Caspase Recruitment Domain-Containing Protein 11 (CARD11) was up-regulated six weeks after PHx, suggesting the involvement of the caspase system at this time. Zinc Finger Protein (ZNF490) gene, with a potential negative effect on cell cycle progression, was only up-regulated at three and six weeks after PHx indicating a central role at this time. TGF-β regulation was not found to be significantly affected in the terminating phase of liver regeneration. Vasohibin 2 (VASH2) was down-regulated towards the end of regeneration, and may indicate a role in preventing a continued vascularization process.

**Conclusions:**

CARD11, ZNF490 and VASH2 are differentially expressed in the termination phase of liver regeneration. The lack of TGF-β up-regulation suggests that signalling by TGF-β is not required for termination of liver regeneration.

## Background

Reestablishment of liver volume after resection is probably regulated by the functional needs of the organism, as the liver regeneration process terminates when the normal liver-mass/body-weight ratio of 2.5% has been restored. A number of studies have been conducted to assess the genetic mechanisms controlling early phases of liver regeneration, mainly in rodents [1-5]. However, the mechanisms controlling the terminating phase have not been investigated to the same extent [6,7].

Two distinct pathways are activated during liver regeneration, the growth factor and cytokine regulated pathways. These regenerative pathways have several checkpoints that could be feedback inhibited and thereby regulate organ size [8]. Amongst cytokines, several negative (Suppressors of Cytokine Signalling (SOCS), IL-6, Plasminogen Activating Inhibitor (PAI)) and positive regulators (Signal Transducer and Activator of Transcription proteins (STAT), Hepatocyte Growth Factor (HGF)) are reported to regulate cell growth [9-11]. Within growth factor pathways, Transforming Growth factor Beta (TGF-β) is a well-known hepatocyte antiproliferative factor. During liver regeneration it has been shown that hepatocytes become resistant to TGF-β and can proliferate despite the presence of TGF-β. SMAD (Small Mothers Against Decapentaplegic) occurs in a downstream signalling pathway of TGF-β. Inhibitors of the TGF-β-SMAD pathway—SKI (Sloan-Kettering Viral Gene Oncolog) and SNON (ski-related novel gene N) are up-regulated during regeneration. SNON and SKI bind SMADs during liver regeneration and might render some cells resistant to TGF-β during the proliferative phase of liver regeneration [12]. However, previous studies have shown that intact TGF-β signalling is not required to stop hepatocyte proliferation once the deficit in liver mass has been replaced [13].

Microarray studies have gained significant importance in experimental research on liver regeneration in recent years. We have shown that the initial regenerative response, quantified by gene expression, was influenced by the grade of resection and the rise in portal pressure [14]. By comparing the findings from that study with the present one, we sought to reveal differences in gene expression in the liver remnant during the initiation and termination of liver regeneration.

After a 70% PHx, the major part of liver regeneration is completed within 7–10 days in the rat and 3 weeks in the pig [15]. Compared to rodents, pigs bear closer genetic and physiological resemblance to man, and we therefore chose to examine this process in the pig. In addition, no previous studies have accounted for the genetic responses in a porcine model in the terminating phase of regeneration.

In this study we aimed primarily to investigate the genetic mechanisms regulating the process of liver regeneration termination in a 60% PHx model in the pig using microarray analysis of gene expression profiles. This was done by 1) classifying all differentially expressed genes by genetic function in order to find genes with specific interest from the beginning of regeneration until the termination phase, 2) by studying the genetic interactions between specific genes regulating the cell cycle, apoptosis and angiogenesis, and 3) by investigating the role of TGF-β signalling in the termination of regeneration, as TGF-β has been proposed to limit the proliferation of hepatocytes [12], but at the same time not to be required to stop hepatocyte proliferation [13].

## Results

### Pigs and surgery

A total of twelve pigs survived the six week experiment, four PHx, four sham operated and four control animals. Pigs that died due to the extensive surgery were replaced: five pigs subject to PHx died, one due to ulcerative gastritis five days post PHx, and one due to blood loss, two days post PHx. Three pigs were terminated, one due to acute pericarditis eight days post PHx, one due to bile-leakage eight days post PHx, and one due to ingestion of foreign materials resulting in occlusion of the oesophagus, 23 days post PHx. One pig subjected to sham operation died due to acute peroperative heart failure during anaesthesia 24 days after primary surgery. All post mortem examinations were performed by an independent official veterinarian at the National Veterinary Institute in Tromsø, Norway.

### Weight and volume of liver at termination

By the end of the sixth week, the liver had fully regenerated in all PHx pigs. In control animals, the liver constituted 2.33% of total body mass, in sham animals the liver constituted 2.48% and in resected animals 2.78% of total body mass.

### Blood sample analysis

We found a significant increase in albumin levels in the sham group at six weeks post PHx. Bilirubin was under the detection level (2.2 mmol/l) for all animals at all time points except in one animal at three weeks with a value of 49 mmol/l. International Normalized Ratio (INR) was less than 1.1 for all animals at all time points. There were no significant time, group or time*group interaction for these analyses.

No significant changes in Interleukin-1 (IL-1), Interleukin-10 (IL-10), Tumor necrosis factor-α (TNF-α) or TGF-β were found. An increase in serum levels of Interleukin-6 (IL-6) was observed in resection group (not significant).

### Microarray analysis

#### General trends

By analysing contrasts between resection, sham and control groups using a false discovery rate (FDR) = 0.20, we found a total of 609 genes differentially expressed (362 genes by comparing control and sham, 215 genes by comparing control and resection, and 32 by comparing sham and resection pigs). Overall, more genes were found associated with the regulation of cell cycle and apoptosis in the liver remnants after PHx compared to livers in the control group. All differentially expressed genes regulating cell cycle and apoptosis are presented in Table 1.

**Table 1:** **Genes proposed to regulate cell cycle and apoptosis with specific functions according to Ace View**[46]

**Resection Group**	**Up-regulated**	**Down-regulated**	**Function**
3-0 weeks	PRKRA (0.8)		Negative regulator of cell proliferation
	GSK3A (0.3)		Negative regulator of cell proliferation
	IGFBP7 (0.9)		Regulation of cell proliferation
		TIA1 (−1.8)	Inducer of apoptosis
6-0 weeks	ZNF490 (2.0)		Negative effect on cell cycle progression and promotes apoptosis
	CCT7 (0.4)		Is implicated in positive control of the G(1)/S phase transition
		BAG3 (−1.1)	Prevents FAS-mediated apoptosis
		TP53INP1 (−0.9)	Induces apoptosis
		TOB (−0.3)	Regulates cell growth
6-3 weeks	ZNF490 (2.4)		Negative effect on cell cycle progression and promotes apoptosis
	CARD11 (0.4)		Activates caspases that play a central role in apoptosis
	PTHLH (0.4)		Positive and negative regulator of cell proliferation
		FAF1 (−1.1)	Increases cell death
**Sham Group**			
3-0 weeks	MDM4 (1.9)		Potentially inhibits the G1 phase of the cell cycle
	E2F2 (0.3)		Helps regulate the expression of a number of genes that are important in cell proliferation
	WWOX (0.2)		Negatively regulates the progression through the cell cycle
	UMOD (0.9)		Negative regulator of cell proliferation
		BRCA1 (−0.6)	Regulate cell-cycle progression, DNA damage repair, cell growth and apoptosis
		SKI (−0.3)	Regulates cell proliferation
6-0 weeks	TPX2 (0.3)		Involved in cellular proliferation
	MDM4 (2.0)		Potentially inhibits the G1 phase of the cell cycle
	CLU (0.4)		Regulates apoptosis
	PROP1 (0.4)		Negatively regulates apoptosis
		CCND2 (−0.3)	May play a distinct role in cell cycle progression
		SOCS2 (−0.9)	Regulates cell proliferation by terminating the transcription activity
6-3 weeks	SKI (0.3)		Regulates cell proliferation
		PECR (−0.5)	Regulates apoptosis
		BTG3 (−0.9)	Is an anti-proliferative gene
**Control Group**			
3-0 weeks	ESR1 (0.6)		Transcription factor binding
		BMP2 (−2.8)	Negatively regulates the progression through cell cycle
		E2F2 (−0.4)	Helps regulate the expression of a number of genes that are important in cell proliferation
		FGF8 (−0.6)	Regulates progression through cell cycle
6-0 weeks	BMPR2 (0.7)		Regulates progression through cell cycle
	CIB1 (0.5)		Signalling cell death
	MPHOSPH9 (0.6)		Regulates progression through cell cycle via M- phase of mitosis
	ELMO1 (0.4)		Promotes phagocytosis, cell shape changes and apoptosis
6-3 weeks	DLEC1 (1.0)		Negatively regulates cell proliferation
		EML4 (−0.3)	Is significantly overexpressed in mitotic cells
		PARD6G (−0.4)	Is involved in cell cycle and cell division

**Figure 1:**
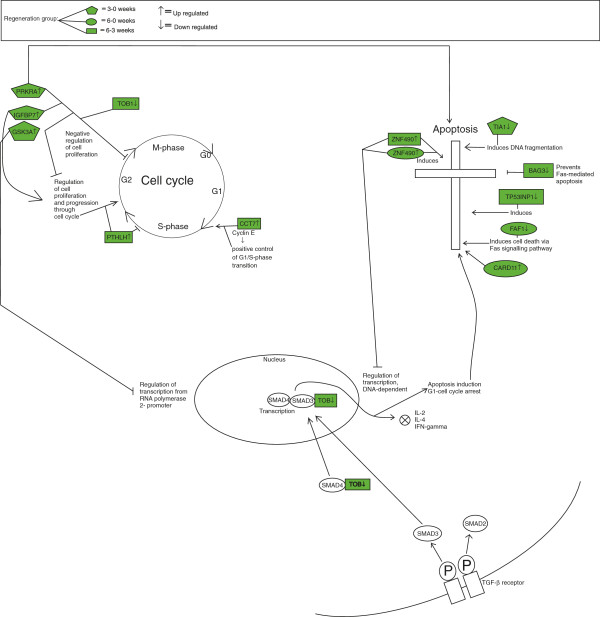
**Differentially expressed genes in resection group at time contrast 3–0, 6–0 and 6–3 weeks. **In resection group, more genes regulate apoptosis towards end of regeneration compared to sham and control group (Figures 2, 3).

**Figure 2:**
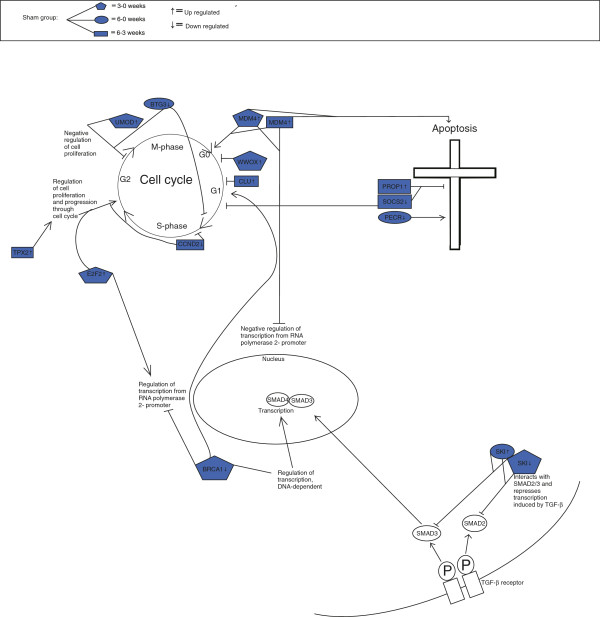
Differentially expressed genes in sham group at time contrast 3–0, 6–0 and 6–3 weeks.

**Figure 3:**
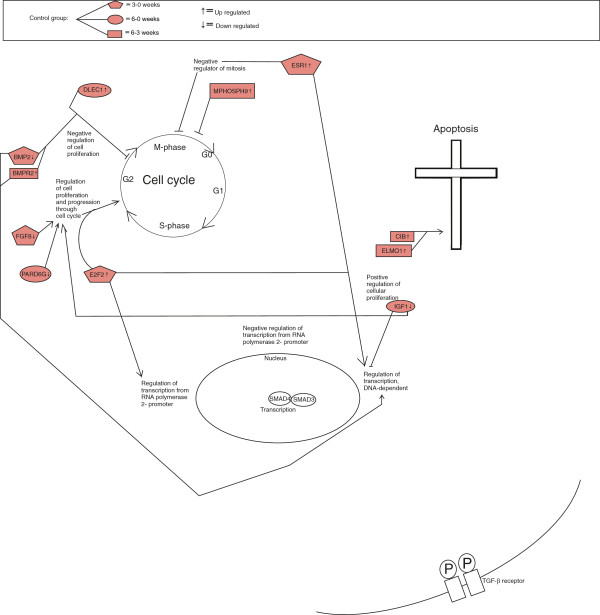
Differentially expressed genes in control group at time contrast 3–0, 6–0 and 6–3 weeks.

When comparing gene expressions at three and six weeks with gene expression at time point 0 weeks, we found the resection group increasingly different over time from both the sham and control group (Figures 1, 2, 3). When comparing the three figures, seven genes were regulating apoptosis in the resection group, whereas only three and two in sham and control group, respectively.

#### General trends of apoptosis, cell cycle and cell proliferation within the resection group

Differentially expressed genes in this chapter are all presented in Figures 1, 2, 3 and Table 1. The text summarizes genes with a log fold change (log FC) over 0.8 in beginning of regeneration, whereas all genes towards termination of regeneration are discussed.

For time contrast 3–0 weeks one gene was up-regulated (log FC 0.9); Insulin-like growth factor binding protein 7 (IGFBP-7). It is involved in regulation of cell proliferation [16]. One gene was down-regulated (log FC −1.8); Cytolytic granule protein (TIA1) which functions potentially as an inducer of apoptosis [17]. For time contrast 6–0 weeks two genes were down-regulated (log FC −1.1): BAG3 potentially prevents FAS-mediated apoptosis [18] while Tumor protein p53 inducible nuclear protein 1 (TP53INP1), (log FC −0.9) potentially induces apoptosis [19].

Towards end of regeneration, one gene found differentially expressed in both time contrasts 6–0 and 6–3 has a potential negative effect on cell cycle progression and promotes apoptosis; Zinc finger protein 490 (ZNF490) [20]. By comparing the log fold change for genes in the resection group, this gene had the highest rate of 2.0 at t = 1, and 2.4 at t = 2. For time contrast 6–3 weeks, one gene was down-regulated (log FC −1.1), that is Fas associated factor 1 (FAF1) which potentially increases cell death [21]. Caspase recruitment domain family, member 11 (CARD11) was up-regulated (log FC 0.4). Parathyroid hormone-like hormone (PTHLH) was also up-regulated in termination of liver regeneration (log FC 0.4), and has been reported to regulate cell proliferation [22].

#### General trends of apoptosis, cell cycle and cell proliferation within the sham group

For time contrast 3–0 weeks, one gene was up-regulated (log FC 0.9): Uromodulin (UMOD) which is a potential negative regulator of cell proliferation [23].

By comparing the first time contrast that is from 0 until 3 weeks, with the second, 6–0, we found one common up-regulated gene, MDM4, (log FC 1.9 and 2.0, respectively). This gene potentially inhibits the G1 phase of the cell cycle [24] in both time-contrasts.

For time contrast 6–0 weeks, one gene regulating cell proliferation was down-regulated: SOCS2 (log FC −0.9). This gene suppresses cytokine signalling and inhibits STAT and thereby terminating the transcription activity [25].

For time contrast 6–3 weeks, one gene was down-regulated, BTG3 (log FC −0.9). This gene is an anti-proliferative gene and ANA is a member of this family. It has been shown that an over expression of ANA impaired serum-induced cell cycle progression from the G0/G1 to S phase [26].

#### General trends of apoptosis, cell cycle and cell proliferation within the control group

For time contrast 3–0 weeks, we found one down-regulated gene (log FC −2.8). Bone morphogenetic protein 2 (BMP2), a member of the transforming growth factor-beta (TGF-β) superfamily, is a potential negative regulator of the progression through cell cycle [27,28].

For time contrast 6–3 weeks, one gene was up-regulated (log FC 1.0). DLEC1, Deleted in lung and esophageal cancer 1, a tumor suppressor gene that may be a potential negative regulator of cell proliferation [29].

#### Top table analysis resection group

**Figure 4:**
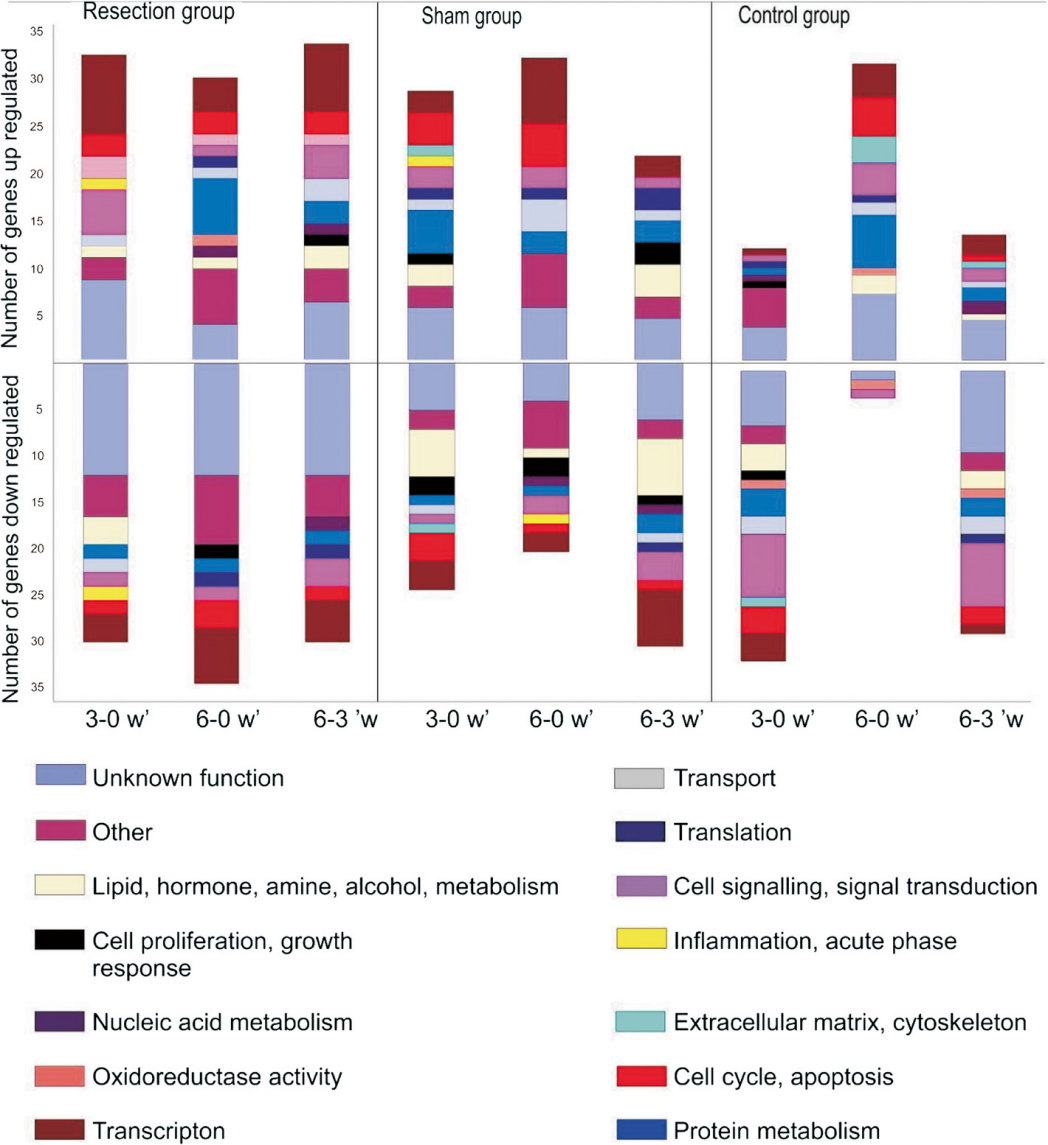
Functional classification of all genes according to Online Mendelian Inheritance in Man and Ace View.

All discussed genes in this chapter are illustrated in Figure 4. Amongst up-regulated genes in the resection group there was in early time period (from t = 0 until t = 1), a predominance of genes regulating transcription, intracellular and cell-cell signalling, extracellular matrix/cytoskeleton and inflammation, whereas genes governing the cell cycle were evenly expressed throughout the experiment. Towards the end of the experiment (from t = 1 until t = 2), we found an increase in up-regulation for genes controlling lipid, hormone, amine, alcohol metabolism and transport.

Amongst down-regulated genes in the resection group there was an increase in number of genes controlling cell cycle and transcription towards the end of the experiment (from t = 1 until t = 2). Genes regulating transport, inflammation and lipid, hormone, amine, alcohol metabolism and transport were only down-regulated in the earliest time period (from t = 0 until t = 1). The expressions of genes regulating cell proliferation were down-regulated at three weeks, whereas genes regulating protein metabolism remained stable. We found a predominance of down-regulated genes regulating intracellular and cell-cell signalling towards the end of liver regeneration.

#### Top table analysis sham group

Amongst up-regulated genes within the sham group, we found from t = 0 until t = 2 a gradual increase in the differential expression of genes controlling cell cycle, transcription and transport. From t = 1 until t = 2, there was a gradual increase in the differential expression of genes governing translation. From t = 0 until t = 1 there was a gradual decrease in expression of genes regulating protein metabolism. In addition, genes regulating intracellular and cell-cell signalling decreased towards the end of the experiment. Genes regulating inflammation and extracellular matrix/cytoskeleton were only up-regulated from t = 0 until t = 1.

Amongst down-regulated genes in the sham group, there was a decrease in down-regulation of genes controlling cell cycle, transcription, transport, extracellular matrix/cytoskeleton and lipid, hormone, amine, alcohol metabolism from t = 0 until t = 1. However, genes controlling transcription, transport, protein metabolism and lipid, hormone, amine, alcohol metabolism increased again towards the end of the experiment. Down-regulated genes controlling intracellular and cell-cell signalling increased in expression from t = 0 until t = 2, whereas genes regulating cell proliferation decreased over all time periods. Genes regulating inflammation were only down-regulated in the middle of the experiment.

#### Top table analysis control group

Amongst up-regulated genes in the control group, the study revealed an increase in expression for genes governing transcription, intracellular and cell-cell signalling and protein metabolism from t = 0 until t = 1, whereas genes regulating translation were evenly expressed in the same period. Genes regulating cell growth were only up-regulated in the early time period. One functional group was only up-regulated at t = 1, genes regulating oxidoreductase activity. Genes regulating nucleic acid metabolism were up-regulated in the beginning and increased towards the end of the experiment. Genes governing transport, protein metabolism, intracellular and cell-cell signalling, cell cycle, extracellular matrix/cytoskeleton, transcription and lipid, hormone, amine, alcohol metabolism decreased in up-regulation from the middle of the experiment towards the end.

Only three functional groups were found at time-contrast two (t = 2); genes with unknown function, genes regulating oxidoreductase activity and genes regulating cell cycle. By comparing the first and the last time contrast (t = 0 versus t = 2), genes regulating oxidoreductase activity, transport and intracellular and cell-cell signalling were evenly expressed. Decreased in down-regulation were genes regulating protein metabolism, cell proliferation, transcription, cell cycle, extracellular matrix/cytoskeleton and lipid, hormone, amine, alcohol metabolism.

#### General trends of angiogenesis and endothelial cell proliferation

**Figure 5:**
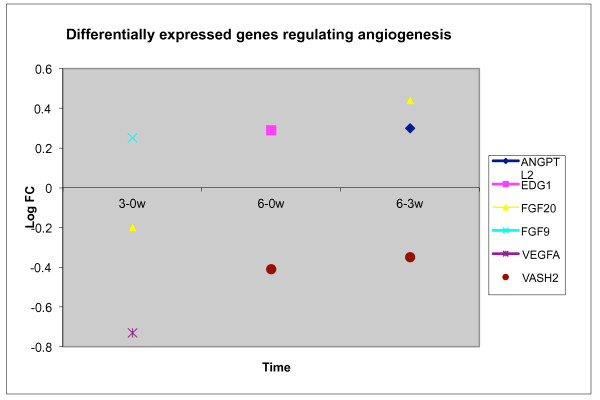
**Differentially expressed genes regulating angiogenesis in resection group.** One gene, VASH2 was downregulated in the middle of and towards the end of regeneration.

In all groups at all time points, 24 genes potentially regulating angiogenesis were differentially expressed, Table 2. In the resection group, seven genes regulating angiogenesis were differentially expressed; three of these towards the end of regeneration. Most genes regulating angiogenesis were differentially expressed in all groups, but one gene was solely expressed in the resection group, Vasohibin 2 (VASH2). This gene positively regulates angiogenesis and positively regulates the proliferation of endothelial cells. VASH2 was down-regulated at both t = 1 and towards the end of regeneration. Figure 5 shows the development over time for genes regulating angiogenesis in the resection group.

**Table 2:** **Genes proposed to regulate angiogenesis with specific functions according to Ace View**[46]

**Resection Group**	**Up-regulated**	**Down-regulated**	**Function**
3-0 weeks	FGF9 (0.3)		Involved in cell growth
		VEGFA (−0,7)	Inducing angiogenesis, vasculogenesis and endothelial cell growth
6-0 weeks	EDG1 (0,3)		Regulate differentiation of endothelial cells
		VASH2 (−0,4)	Positive regulation of angiogenesis and endothelial cell proliferation
6-3 weeks	ANGPTL2 (0,3)		Growth factor specific for vascular endothelium
	FGF20 (0,4)		Involved in cell growth
		VASH2 (−0,3)	Positive regulation of angiogenesis and endothelial cell proliferation
**Sham Group**			
3-0 weeks	ANGPTL3 (0,2)		Growth factor specific for vascular endothelium
		ANGPT2 (−0,2)	Negative regulation of angiogenesis by inducing endothelial cell apoptosis
6-0 weeks	FAP (0,2)		Involved in control of fibroblast growth
	FGF9 (0,3)		Involved in cell growth
	FGFBP3 (0,3)		Positive regulation of fibroblast growth factor
	VEZF1 (0,8)		Participates in angiogenesis
6-3 weeks	VEZF1 (1,0)		Involved in angiogenesis
	VEZF1 (0,7)		Involved in angiogenesis
		AMOTL2 (−0,2)	Angiomotin binds angiostatin, an inhibitor of angiogenesis
		FGFR1OP (−0,2)	Involved in angiogenesis and cell growth
**Control Group**			
3-0 weeks	AMOTL1 (0,4)		Angiomotin binds angiostatin, an inhibitor of angiogenesis
		FGF8 (−0,6)	Involved in cell growth
6-0 weeks	AMOTL1 (0,7)		Angiomotin binds angiostatin, an inhibitor of angiogenesis
	FGF20 (0,4)		Involved in cell growth
		FGFR3 (−0,2)	Involved in cell growth
6-3 weeks	FGF8 (0,4)		Overexpression has been shown to increase tumor growth and angiogenesis
		VEZF1 (−0,9)	Involved in angiogenesis

## Discussion

In this study we aimed to investigate genes regulating the terminal phase of liver regeneration, to illuminate the genetic interactions between genes controlling cell cycle, apoptosis and angiogenesis, and to clarify the role of TGF-β signalling in the termination of liver regeneration.

Analysis of the microarray data shows several trends governing the termination of the regeneration process in the liver. As expected, more genes were found associated with the regulation of the cell cycle and apoptosis when comparing gene expression in the biopsies from the regenerating livers, to the liver biopsies from control animals (Figures 1, 2, 3). On the other hand, it is interesting to observe that several other genes with similar functions are differentially expressed in the sham and control groups. This in turn, is tentatively an indication of the fact that the normal growing, non-resected liver is under constant control by the opposing actions of pro-mitotic and pro-apoptotic genes and their protein products, maintaining a constant liver weight/body mass ratio and metabolic function as required.

Secondly, more genes were differentially expressed in the time contrast 6–3 weeks in the resection group compared with the sham and control group (Table 1). This is probably a reflection of the fact that the regenerating liver is genetically more active not only after a resection as compared to sham and control livers, but it also indicates that the regenerative response continues for many weeks.

Thirdly, for both comparisons in the contrasts of contrasts analysis, we observed a tendency of increasing differences in gene expression between the regenerating livers and the sham and control livers over time. A natural interpretation of this observation could be that, as the postoperative acute phase reaction subsides; prominent genetic patterns governing regeneration come to surface, some of which are shown in the present study.

With regard to established “stop” signals of hepatocyte proliferation and liver regeneration, this study can only partly corroborate the conclusions of most previous studies. We can however, report the “finding” of genes associated with genes known to interact with cell cycle propagation and apoptosis. For instance, TGF-β was not found in our material. However, TOB1 (Transducer of ERBB2, 1), a down regulated gene in regenerating livers, has been reported to bind SMAD4 (Small Mothers Against Decapentaplegic) and thereby render some cells resistant to TGF-β [30,31]. This gene occurred in the resection group at time-contrast 6–0, indicating a down-regulation of its antiproliferative property in the middle of the experiment. At the same time, the TOB1-SMAD4 complex inhibits IL-2, IL-4 and Interferon-gamma-γ (IFNγ) and induces apoptosis and G1 cell cycle arrest in hepatocytes [30]. SKI (Sloan-Kettering Viral Gene Oncolog) was down-regulated in early phase of sham group, indicating an inactivation of SMAD-binding, thereby admitting TGF-β’s antiproliferative function. Another gene, BMP2 (Bone Morphogenetic Protein 2), a member of the TGF-β-superfamily, was down-regulated in the control group during the early time period. TGF-β has been shown to orchestrate multiple events as part of a large feedback loop during regeneration [31] and our findings (TOB1, SKI and BMP2) is in line with previous studies, but without a direct involvement of TGF-β. This again, is in accordance with the findings from Oe et al., concluding that intact signalling by TGF-beta is not required for termination of liver regeneration [13]. They suggest that an increase of activin A signalling may compensate to regulate liver regeneration when signalling through the TGF-β pathway is abolished, and may be a principal factor in the termination of liver regeneration [13]. In our opinion, the findings of TOB1, SKI and BMP2 adds credibility to our study, at the same time as the lack of TGF-β support the findings from Oe et al. [13].

In the resection group, we observed a pattern for differentially expressed genes regulating cell cycle and apoptosis, as three out of four genes in the early time phase of regeneration regulated the cell cycle, whereas towards the end of the experiment, seven out of ten genes regulated apoptosis. This suggests an initiating event of up-regulated cell cycle genes, as well as a termination phase governed by apoptotic genes. However, some of these genes had an inhibitory function of both cell cycle and apoptosis, indicating constant control by the opposing actions of pro-mitotic and pro-apoptotic genes. A small wave of apoptosis of hepatocytes seen at the end of DNA synthesis suggests that this is a mechanism to correct an over-shooting of the regenerative response [32]. Specifically, we observed in the resection group that genes promoting apoptosis and inhibiting cell cycle, like ZNF490 and CARD11 were up-regulated towards the end of the experiment, suggesting a crucial role of these genes at this time. In addition, genes regulating apoptosis in the middle of the experiment were both down- and up-regulated, indicating a complex process before termination of regeneration. Within the sham and control group at the end of the experiment, three and four genes regulated apoptosis, respectively. From these results, it seems as if the gene expression in the resection group was more focused towards apoptotic function compared to sham and control group (Figures 1, 2, 3).

Functional classification of the differentially expressed genes with Ace View and OMIM demonstrates the complexity of the genetic response over time in the three groups, as genes representing almost all functional groups are differentially expressed at one time or another. This has been shown in previous studies dealing with liver regeneration, and is not surprising, as the process of liver regeneration involves multiple metabolic pathways [33]. Interestingly, in the resection group overall more genes regulate transcription, nearly twice as many as in control group, suggesting an explanation of the rapid growth of the regenerating liver. There was also a clear dominance in the amount of genes regulating cell cycle and apoptosis towards the end of regeneration in the resection group, Figure 2. This adds credibility to the above mentioned mechanism of over-shooting of the regenerative response [32].

With regard to Top table analysis, we observed several patterns within the respective groups. Specifically, we observed in the resection group a predominance of up-regulated genes regulating transcription, cell signalling, extracellular matrix and inflammation in earlier time periods, suggesting a complex process after PHx with a combination of inflammation and induction of regeneration. In contrast to the sham group, genes governing cell cycle in the resection group were evenly expressed throughout the experiment, indicating a constant regulation of cell proliferation during regeneration. In addition, we found in the resection group that genes regulating protein- and nuclear acid metabolism were up-regulated at three weeks and in the end of regeneration, tentatively due to the need of nuclear acids in DNA-synthesis as the liver regenerates.

As described, we observed in the early phase of regeneration, a predominance of genes governing transcription. Of seven up-regulated genes in the early time phase for the resection group, four were members of the zinc finger protein family. Previous studies report that some zinc finger genes function as transcriptional repressors [34], while other that zinc-finger proteins (ZFPs) function as sequence-specific DNA-binding transcription factors, with important roles in a variety of biological processes, such as development, differentiation, and tumor suppression [35], which might be of significant importance in the beginning of regeneration as these factors initiates genes necessary for cell division and cell growth.

In the early time period of regeneration (0–3 weeks), some genes could in theory have a positive effect on hepatocyte proliferation, for instance Fas apoptotic inhibitory molecule 2 (FAIM2). An up-regulation of these genes may suggest the rapid cell growth of hepatocytes after PHx. On the other hand, we observed an up-regulation of genes negatively regulating cell cycle at the end of regeneration (6 weeks). CARD11 is a gene involved in assembly of signal complexes leading to activation of caspase family. Caspases are cysteine proteases that play a central role in apoptosis [36], suggesting a negative regulatory function in the end of regeneration. The down-regulation of IGFBP7 after three weeks is a possible commencement of growth restriction already at this time.

Recently, some studies have described Micro-RNAs (miRNAs) as modulators of liver regeneration termination [37,38]. There were no known genes differentially expressing miRNAs in our material.

Little has been documented about genes regulating angiogenesis in the termination of liver regeneration. We sought to investigate genes regulating angiogenesis towards the end of regeneration. One gene, VASH2, was only expressed in the resection group. Expression of this gene leads to angiogenesis [39]. Interestingly, this gene was down-regulated at both three weeks and towards the end of regeneration. Inhibition of this gene might play a role preventing a continued vascularization process.

## Conclusions

Our data reveal the following genetic regulation in liver regeneration termination: 1) Caspase Recruitment Domain-Containing Protein 11(CARD11) gene, involved in assembly of signal complexes leading to activation of caspase family and apoptosis was up-regulated six weeks after liver resection, suggesting the involvement of the caspase system at this time; 2) Zinc Finger Protein (ZNF490) gene, with a potential negative effect on cell cycle progression and promotion of apoptosis, was up-regulated at three and six weeks after resection, and may indicate a central role in the regulation of liver regeneration termination; 3) Vasohibin 2 (VASH2) gene, regulates angiogenesis and positively regulates the proliferation of endothelial cells. It was down-regulated at both three weeks and towards the end of regeneration, suggesting a role in preventing a continued vascularization process; 4) The lack of TGF-β gene expression and ELISA confirms the findings from Oe et. al. [13], verifying the assumption that intact signalling by TGF-β is not required for termination of liver regeneration.

## Methods

### Experimental setup

Twelve female Norwegian landrace pigs, weighing 31.7 (± 5.13) kg from a single commercial farm were used. The animals were housed in a closed-system indoor facility with 55 ± 10% relative humidity, 17–18 air changes per hour and temperature of 20 ± 1°C. The pigs shared fenceline contact with another related pig and were singly housed in 1.5 × 1.5 m pens with ad libitum access to tap water from water nipples, liquid dietary supplement and digestive energy mixed with water. Light was supplied on a 12:12 hour schedule.

Four pigs were subject to a 60% PHx (group one), four pigs were subject to sham surgery (group two) and four pigs were used as controls (group three). Control animals were necessary, as all of these animals were growing, and a measurement of normal liver growth was needed. All pigs were re-operated at three- and at six weeks post PHx. Biopsies were sampled upon initial laparotomy (t = 0), at three weeks post PHx (t = 1) and upon termination at six weeks post PHx (t = 2).

This project was approved in agreement with the Norwegian Animal Welfare Act § 21 and The Norwegian Regulation on Animal Experimentation §§ 7, 8 and 13. Our department is run in agreement with the European Convention for the Protection of Vertebrate Animals used for Experimental and Other Scientific Purposes.

### Anaesthesia

The animals were fasted overnight with free access to water. They were initially sedated with Ketamin (10 mg/kg intramuscularly (i.m.)) and Atropin (0.05 mg/kg i.m.). All animals were intubated, and anaesthesia was maintained with Isoflurane 1.5–2% mixed with 50–60% oxygen. Respiratory rate was adjusted to achieve an Et CO_2_ between 35 and 40 mmHg. Intravenous (i.v) access was obtained through a vein on the ear. Analgesia was induced and maintained with Fentanyl 0.01 mg/kg, i.v. All animals received a peroperative i.v. volume load consisting of 1000 ml Ringer solution. Volume infusion was continued thereafter with 20 ml/kg/hr 0.9% NaCl and 10% Glucose. Before surgery, all animals received a single intramuscular injection of antibiotic prophylaxis with Enrofloxacin 2.5 mg/kg.

### Monitoring

The cardio-respiratory status was monitored with an electrocardiogram (ECG), invasive arterial blood pressure via a cannula in the femoral artery and by hourly arterial blood gas analysis. Intravascular pressure monitoring was performed using calibrated transducers connected to an amplifier (Gould, 2800S, Ohio, USA). Portal venous pressure was monitored via a paediatric central venous catheter (CVK (Arrow International)) placed directly in the portal vein. Mean alveolar concentration of Isoflurane was monitored using a Capnomac (Nycomed Jean Mette). Body temperature was maintained at approximately 39°C with a heating blanket. All recordings were documented hourly until extubation. The same anaesthesia protocol was employed for surgery at 3 and 6 weeks after PHx.

Upon experiment termination, the pigs were sacrificed with an overdose of 100 mg Pentobarbital i.v. and 20 mmol KCl intracardially. The liver was removed and volume and wet weight was measured.

### Surgical procedures

A midline laparotomy was used for access to the hepatic hilus. A reference biopsy was sampled from segment IV before resection (t = 0) and stored immediately in RNALater (Ambion).

Blood extraction was performed via a Hickman catheter (BARD Access Systems) placed in the Jugular vein. This access was also used for blood sampling and postoperative administration of intravenous fluids and medication. A Freka Percutaneous Enteral Gastrostomy (PEG, Fresenius Kabi AG) was placed in the stomach to prevent gastric retention, observed in pilot experiments. The hepatic artery supplying segments II and III together with these segments’ portal branch were ligated using an absorbable polyfilament suture on a large needle. Thereafter the lobe was strangulated with a 0.5 cm wide cotton ribbon and then removed and weighed. Segments IV, V and VIII were removed in a similar manner leaving segments VI, VII and I in place corresponding to an approximate 60% PHx.

In group two (sham), the pigs underwent a midline laparotomy, biopsy of segment IV, placement of the Hickman catheter in the Jugular vein and placement of the Freka Percutaneous Enteral Gastrostom (PEG, Fresenius Kabi AG). That is, the exact same procedure as in resected animals, except liver resection. In group three (control), the pigs underwent a minimal laparotomy for biopsy sampling from segment IV. Blood was sampled from the jugular vein. No catheters were used.

### Recovery

Postoperative pain management was maintained with a transdermal Fentanyl patch (Hexal A/S) delivering 50 μg/72 h, exchanged with a patch delivering 25 μg/72 h Fentanyl the following three days. All pigs received water ad libitum and 3 dl of liquid dietary supplements four times per day the first postoperative week, together with a standardized amount of solid pig-feed amounting to 2546 Kcal per day. I.v. fluids were administered daily via the Hickman catheter in the right Jugular vein for pigs in group one and two. The first week the pigs received 250 ml 5% Glucose (Fresenius Kabi AB) mixed with 20 mg Esomeprazol (Astra Zeneca) in the morning, 500 ml Ringer’s solution (Baxter Medical AB) mixed with 50 mg Erytromycin (Abbott Scandinavia AB) at noon, and 250 ml 5% Glucose mixed with 20 mg Esomeprazol in the afternoon. Extended i.v. Glucose infusion (500 ml 5% glucose) was given when the animals in the resection group suffered of anorexia postoperatively. Oral medication was continued with 5 mg/kg Erytromycin daily and 20 mg Esomeprazol twice daily, until biopsy three weeks post PHx. After biopsy the third week, the pigs in group one and two again received i.v. fluids via a new Hickman catheter placed in the left jugular vein. The same amount of fluids and medication was given at the same time each day as after primary operation, but only for three days postoperatively. Oral medication was continued with 5 mg/kg Erytromycin daily and 20 mg Esomeprazol two times per day, until sacrificing the sixth week.

### Blood sampling

For pre-PHx reference values, blood was sampled from the jugular vein at the time of laparotomy. After surgery, we sampled regularly from the jugular vein for analysis of: 1) Cytokines: *IL-1, IL-6, IL-10* (Multiple cytokine analyses (Multiplex®, Tromsø, Norway); 2) Humoral growth regulating factors: *TNF-α* (Multiple cytokine analyses (Multiplex®, Tromsø, Norway), *TGF-β* (MILLIPLEX MAP TGF ß1 (Transforming Growth Factor Beta) - Single Plex, Tromsø, Norway).

### Other analysis

*ASAT, ALAT, γGT* (Roche/Hitachi, enzymatic colometric assay. Reagent: Mannheim, Germany. Chemistry analyzer: Roche diagnostics, Hitachi, Japan); *Bilirubin, Albumin* (Roche/Hitachi, colometric assay. Reagent: Mannheim, Germany. Chemistry analyzer: Roche diagnostics, Hitachi, Japan)

*INR* (STA - SPA 50 kit, STA-R, Diagnostika Stago- 9, Asnieres, France)

### Statistics

Time, group and group*time interaction of blood analyses was examined using General Linear Model with Repeated Measures in SPSS version 15, with p ≤ 0.05 considered significant. We defined time as a fixed factor and subject as a random effect. An autoregressive AR1 covariance matrix was used. All curves for all animals in all groups are drawn as group averages ± 1 SD.

### Biopsies

A reference sample was taken from all animals in all groups upon laparotomy, before PHx (t = 0), at time points three weeks post PHx (t = 1) and six weeks post PHx (t = 2). Biopsies were immersed immediately in RNAlater (Ambion®), and preserved at – 70°C until RNA extraction and microarray analysis.

### Microarray methods

Two-colour microarray experiments were conducted to identify genes being significantly differentially expressed due to resection over time adjusting for effects by using the expression profiles obtained from the control animals and the sham operated animals.

The microarray experiment was conducted as a common reference design using a reference consisting of equal amounts of total-RNA from all samples. Total-RNA was extracted from each sample and DNase treated using RNeasy Maxi Kit (Qiagen). Quantities were measured using a NanoDrop ND-1000 Spectrophotometer (NanoDrop Technologies, DE, USA) and qualities were examined by the 28S:18S rRNA ratio using the RNA 6000 Nano LabChip® Kit on 2100 Bioanalyzer (Agilent Technologies, CA, USA). Alexa Flour-labeled cDNA was synthesized from 20 μg of total-RNA using Superscript Plus Direct cDNA Labeling System (Invitrogen) and purified using the NucleoSpin 96 Extract II PCR Clean-up kit (Macherey-Nagel, Düren, Germany). The reference samples were labelled with Alexa-555 and the individual samples were labelled with Alexa-647. The labelled and purified reference samples were mixed and divided into aliquots before combining it with a labelled sample. Each of the 36 labelled samples were co-hybridized with an aliquot of the labelled reference sample and a hybridization blocker containing polydA (Invitrogen Corporation, CA, USA) and Yeast tRNA (Invitrogen Corporation, CA, USA) to 27k pig oligonucleotide microarrays representing approximately 20k porcine genes using a Discovery XT hybridisation station (Ventana Discovery Systems, Illkirch CEDEX, France). Detailed description of the microarray used in this study can be found at NCBI’s Gene Expression Omnibus (GEO, [40,41]http://www.ncbi.nlm.nih.gov/geo) using the accession GPL5972.

Following hybridization, washing and drying, the slides were scanned in a ScanArray Express HT system (version 3.0, Perkin Elmer, Hvidovre, Denmark) and the resulting images were analyzed using GenePix Pro (version 6.1.0.4, Molecular Devices). Statistical analysis was carried out in the R computing environment (version 2.6.1 for Windows) using the package Linear Models for Microarray Analysis (Limma, version 2.12.0, [42]) which is part of the Bioconductor project [43]. Spots marked as “Not found” by GenePix and spots with more than 50% of saturated pixels were weighted “0” before the log_2_-transformed ratios of Alexa-647 to Alexa-555 (not background corrected) were normalized within-slide using global-loess with default parameters as implemented in Limma. The set of normalized log-ratios were then analyzed in Limma to identify genes being significantly differentially expressed due to resection over time adjusting for effects by using the expression profiles obtained from the control animals and the sham operated animals. The false discovery rate was controlled using the method of Benjamini and Hochberg [44] as implemented in Limma and a corrected P-value below 0.20 was considered significant. A detailed description of the microarray experiment together with the resulting dataset is available at NCBI’s Gene Expression Omnibus (GEO, [40,41]http://www.ncbi.nlm.nih.gov/geo) using the accession number GSE14396.

According to OMIM [45] and Ace View [46], we classified all top 50 genes into 14 groups by molecular function and biological process. First, this functional classification was illustrated by using top tables for each time contrast (3–0 weeks, 6–0 weeks and 6–3 weeks). Second, this set of genes was further analyzed by finding genes associated with genes regulating cell cycle propagation and apoptosis that we previously found in an acute model of liver resection [14]. Third, to highlight differences in temporal differential gene expression between groups “contrast of contrast” analyzes was conducted. According to Wack et al. [47] proliferation and migration of the sinusoidal endothelium into the avascular hepatic islands is suspected to be driven by the up-regulation of various angiogenic growth factors. Using the stepwise approach described above (1 and 2), we sought and analyzed genes associated with angiogenesis and endothelial cell proliferation at all time points.

## Abbreviations

(BMP2): Bone morphogenetic protein 2; (CARD 11): Caspase Recruitment Domain-Containing Protein 11; (CVK): Central venous catheter; (TIA1): Cytolytic granule protein; (DLEC1): Deleted in lung and esophageal cancer 1; (ECG): Electrocardiogram; (ELISA): Enzyme-linked Immunosorbent Assay; (FAIM2): Fas apoptotic inhibitory molecule 2; (FAF1): Fas associated factor 1; (HGF): Hepatocyte Growth Factor; (IGFBP-7): Insulin-like growth factor binding protein 7; (IFNγ): Interferon-gamma-γ; (IL): Interleukin; (INR): International Normalized Ratio; (i.m.): Intramuscularly; (i.v): Intravenous; (miRNAs): Micro-RNAs; (PHx): Partial hepatectomy; (PEG): Percutaneous Enteral Gastrostomy; (PAI): Plasminogen Activating Inhibitor; (PRKRA): Protein kinase; (STAT): Signal Transducer and Activator of Transcription proteins; (SKI): Sloan-Kettering Viral Gene Oncolog; (SMAD): Small Mothers Against Decapentaplegic; (SNON): Ski-related novel gene N; (SOCS): Suppressors of Cytokine Signalling; (TOB1): Transducer of ERBB2; (TGF-β): Transforming Growth Factor-β; (TNF-α):Tumor necrosis factor-α; (TP53INP1): Tumor protein p53 inducible nuclear protein 1; (UMOD): Uromodulin; (VASH2): Vasohibin 2; (ZNF490): Zinc Finger Protein.

## Competing interests

The authors declare that they have no competing interests.

## Authors’ contributions

IEN authored the study protocol, performed all surgical experiments, interpreted all results drafted and revised the manuscript. KEM has made substantial contribution in conduction of the liver surgery and has been involved in revising the manuscript for important intellectual content. JH, LNC and CB was responsible for all aspects of the microarray analysis, performed the statistical analysis and have been involved in drafting the manuscript. TK carried out the cytokine analysis. AR conceived of the study, participated in its design and coordination and helped to draft the manuscript. All authors read and approved the final manuscript.

## Authors’ information

IEN: Resident at the Department of Digestive Surgery, University Hospital of Northern Norway, Tromsø, Norway. KEM: PhD, Department of Digestive Surgery, University Hospital of Northern Norway, Tromsø, Norway. JH: PhD, Institute of Clinical Medicine, Department of Molecular Medicine, Aarhus University Hospital, Aarhus, Denmark. LNC: PhD, Department of Genetics and Biotechnology, Faculty of Agricultural Sciences, University of Aarhus, Denmark. TK: Head Engineer at the Laboratory of Surgical Research, Institute of Clinical Medicine, University of Tromsø, Norway. CB: Professor at the Department of Genetics and Biotechnology, Faculty of Agricultural Sciences, University of Aarhus, Denmark. AR: Professor at the Laboratory of Surgical Research, Institute of Clinical Medicine, University of Tromsø, Norway.
